# Single Nucleotide Polymorphisms in the Wnt and BMP Pathways and Colorectal Cancer Risk in a Spanish Cohort

**DOI:** 10.1371/journal.pone.0012673

**Published:** 2010-09-09

**Authors:** Ceres Fernández-Rozadilla, Luisa de Castro, Juan Clofent, Alejandro Brea-Fernández, Xavier Bessa, Anna Abulí, Montserrat Andreu, Rodrigo Jover, Rosa Xicola, Xavier Llor, Antoni Castells, Sergi Castellví-Bel, Angel Carracedo, Clara Ruiz-Ponte, for the Gastrointestinal Oncology Group of the Spanish Gastroenterological Association

**Affiliations:** 1 Galician Public Foundation of Genomic Medicine (FPGMX), Centro de Investigación Biomédica en Red de Enfermedades Raras (CIBERER), Genomics Medicine Group, Hospital Clínico, Santiago de Compostela, University of Santiago de Compostela, Galicia, Spain; 2 Gastroenterology Department, Hospital Meixoeiro, Vigo, Galicia, Spain; 3 Gastroenterology Department, Hospital La Fe, Valencia, Spain; 4 Gastroenterology Department, Hospital del Mar, Institut Municipal d'Investigació Médica (IMIM), Pompeu Fabra University, Barcelona, Catalonia, Spain; 5 Unidad de Gastroenterología, Hospital General Universitario de Alicante, Alicante, Spain; 6 Section of Digestive Diseases and Nutrition, University of Illinois at Chicago, Chicago, Illinois, United States of America; 7 Department of Gastroenterology, Hospital Clínic, CIBERehd, IDIBAPS, University of Barcelona, Barcelona, Catalonia, Spain; University of Hong Kong, Hong Kong

## Abstract

**Background:**

Colorectal cancer (CRC) is considered a complex disease, and thus the majority of the genetic susceptibility is thought to lie in the form of low-penetrance variants following a polygenic model of inheritance. Candidate-gene studies have so far been one of the basic approaches taken to identify these susceptibility variants. The consistent involvement of some signaling routes in carcinogenesis provided support for pathway-based studies as a natural strategy to select genes that could potentially harbour new susceptibility loci.

**Methodology/Principal Findings:**

We selected two main carcinogenesis-related pathways: Wnt and BMP, in order to screen the implicated genes for new risk variants. We then conducted a case-control association study in 933 CRC cases and 969 controls based on coding and regulatory SNPs. We also included rs4444235 and rs9929218, which did not fulfill our selection criteria but belonged to two genes in the BMP pathway and had consistently been linked to CRC in previous studies. Neither allelic, nor genotypic or haplotypic analyses showed any signs of association between the 37 screened variants and CRC risk. Adjustments for sex and age, and stratified analysis between sporadic and control groups did not yield any positive results either.

**Conclusions/Significance:**

Despite the relevance of both pathways in the pathogenesis of the disease, and the fact that this is indeed the first study that considers these pathways as a candidate-gene selection approach, our study does not present any evidence of the presence of low-penetrance variants for the selected markers in any of the considered genes in our cohort.

## Introduction

Colorectal cancer (CRC) is one of the main forms of cancer, being the second most frequent neoplasm in both sexes and one of the most important morbidity causes in the western world [1]. The genetic contribution to CRC has been estimated to be around 35% by extensive twin studies [2]. However, highly penetrant variants, that cause mendelian predisposition syndromes, account only for, at most, 5% of the disease cases [3]. The remaining genetic susceptibility is thought to follow a polygenic model, with an interplay of multiple low-penetrance allelic variants appearing in high frequency in the general population, and each conferring a modest effect on disease risk [4], [5].

Candidate-gene studies have been one of the most commonly used tools in the screening for new variants affecting CRC risk. Gene selection in these studies is mainly based on the functional implications of a possible association, and thus genes selected have either been chosen because of the previous presence of other high/low risk alleles [6], or their participation in a pathway implicated in the pathogenesis of the disease [7]. Candidate-gene studies can be performed by either direct approaches, where the variants genotyped are presumed to be the underlying cause of the disease because of their location (variants in exonic or regulatory regions), or by indirect approaches, where tag SNPs take advantage of the linkage disequilibrium properties of the human genome to try and screen the most of the variability in a given gene.

This latter approach has also allowed, together with the development of high-throughput technologies, the implementation of new hypothesis-free approaches (in opposition with hypothesis-based candidate-gene approaches), covering the majority of the genome (genome-wide association studies or GWAS). This implementation has successfully led to the identification of some new susceptibility loci [8]–[14], including rs4444235 and rs9929218, that fall within reach of two genes belonging to the BMP pathway. Nevertheless, these have been found to predict only a small proportion of the disease susceptibility, with the remaining yet to be discovered [15].

We hence aimed to find such susceptibility variants through a candidate-gene approach screening a selected number of variants within two cellular pathways that have consistently been linked to CRC tumorogenesis: the Wnt and the BMP signaling pathways [16], [17].

The Wnt pathway contains genes that have for long been known to be responsible of some hereditary CRC syndromes, such as *APC* and familial adenomatous polyposis [18]. Moreover, somatic alterations in *APC* are found in almost 80% of the sporadic colorectal cancers, and Wnt signaling activation is involved in the best part of sporadic colorectal carcinomas [19]. On the other hand, the BMP pathway acts as positive regulator of some of the Wnt proteins [17], and the tumor suppressive role of this signaling pathway in the pathogenesis of CRC and other cancers is well established [20], [21]. Besides, mutations in two of its genes, *SMAD4* and *BMPR1A*, are responsible for juvenile polyposis syndrome, another hereditary CRC condition [22]. Considering all this information, we thought it would be interesting to screen some of the genetic variability within these pathways for any evidence of new CRC related variants that could explain at least part of the missing heritability. Our approach was mainly functional, for only SNPs within exonic or *cis*-regulatory sequences (5′ and 3′ unstranslated regions) were selected to analyse their relationship with CRC susceptibility.

## Results and Discussion

Following our pathway-based candidate-gene selection method, we performed our study in a total of 45 SNPs that were in either exonic or regulatory regions, in an overall of 21 genes from both the Wnt and BMP pathways. Details of SNP features and association values for the 37 SNPs that successfully passed quality control criteria are shown on Table 1. None of the screened SNPs were significantly associated with an altered risk of CRC, considering odds-ratios and related p values for allelic and genotypic tests (trend, dominant and recessive). Logistic regression for age and sex adjustment was performed, although it did not improve p value results. Haplotype analysis results were consistent in both Unphased and Haploview, and did not show any signs of positive associations either for any of the 8 genes for which this analysis was performed (*AXIN1*, *HDAC9*, *BMP4*, *DACT1*, *CDH3*, *CDH1*, *BTRC*, and *APC)*, (Figure S1). Stratification analysis comparing sporadic and familial cases was also implemented, but it did not provide any evidence of differences in susceptibilities between the groups that could be a sign of any specific associations within either of the groups (Table 2).

**Table 1:** Description of the 37 SNPs that passed quality control criteria and their associated p values.

Gene	SNP ID	SNP type	Amino acid change	Allele	MAF cases	MAF controls	GT counts cases	GT counts controls	p-value	OR (95% CI)
*ADAR*	rs2229857	Missense	K384R	**A**/G	0.3306	0.3201	99/360/385	88/347/382	0.512	1.05 (0.91–1.22)
*APC*	rs2229992	Synonymous	Y486Y	C/**T**	0.3981	0.4125	145/382/317	141/392/284	0.3728	0.94 (0.82–1.08)
*APC*	rs351771	Synonymous	A545A	**C**/T	0.3817	0.375	124/397/324	125/416/347	0.7978	1.02 (0.89–1.18)
*APC*	rs41115	Synonymous	T1493T	**C**/T	0.3796	0.3761	126/385/328	127/414/347	0.8595	1.00 (0.88–1.16)
*APC*	rs42427	Synonymous	G1678G	A/**G**	0.3741	0.3713	124/382/336	116/365/323	0.9252	1.01 (0.88–1.17)
*APC*	rs459552	Missense	V1822D	**A**/T	0.2302	0.2134	48/293/504	41/297/550	0.2197	1.11 (0.94–1.30)
*APC*	rs465899	Synonymous	P1960P	**C**/T	0.3828	0.3743	126/395/324	125/414/348	0.7107	1.03 (0.90–1.19)
*APC*	rs866006	Synonymous	S1756S	**A**/C	0.3775	0.3756	123/370/323	124/401/339	0.925	1.00 (0.87–1.19)
*AXIN1*	rs1805105	Synonymous	D254D	C/**T**	0.3918	0.4096	136/387/318	164/397/324	0.2692	0.93 (0.81–1.07)
*AXIN1*	rs214250	Synonymous	S428S	C/**T**	0.2206	0.2028	32/307/502	34/265/522	0.2138	1.12 (0.94–1.32)
*AXIN1*	rs214252	Synonymous	A609A	A/**G**	0.2207	0.2005	32/305/499	34/258/521	0.1403	1.13 (0.96–1.34)
*AXIN1*	rs400037	Missense	R388Q	C/**T**	0.1826	0.1829	27/244/545	39/234/580	0.8972	1.04 (0.87–1.24)
*AXIN2*	rs2240308	Missense	P50S	**A**/G	0.4502	0.4219	168/423/252	152/442/290	0.1031	1.11 (0.97–1.27)
*BMP4*	rs17563	Missense	V152A	C/**T**	0.4946	0.4855	211/407/220	208/420/233	0.5498	1.07 (0.93–1.23)
*BMP4*	rs4444235	–	–	C/**T**	0.4563	0.4557	168/436/242	196/411/274	0.9343	0.99 (0.86–1.14)*
*BTRC*	rs17767748	Synonymous	I229I	C/**T**	0.05516	0.056	3/86/745	4/91/789	0.9324	1.00 (0.74–1.36)
*BTRC*	rs4151060	Missense	A543S	G/**T**	0.04793	0.04904	4/73/768	2/83/802	0.6997	0.96 (0.70–1.32)
*CCND1*	rs603965	Synonymous	P241P	**A**/G	0.4969	0.4822	204/406/209	206/430/237	0.4164	1.06 (0.93–1.22)
*CDH1*	rs1801552	Synonymous	A692A	C/**T**	0.3547	0.3781	105/371/343	126/365/325	0.1834	0.92 (0.81–1.07)
*CDH1*	rs9929218	Intronic	–	**A**/G	0.2811	0.2873	65/345/435	83/342/459	0.5486	0.97 (0.83–1.13)*
*CDH3*	rs1126933	Missense	Q563H	**C**/G	0.3828	0.3802	129/382/325	129/361/324	0.8369	1.02 (0.88–1.17)
*CDH3*	rs17715450	Synonymous	R747R	A/**C**	0.3783	0.3959	116/390/316	147/402/330	0.2792	0.93 (0.80–1.07)
*CDH3*	rs2274239	Synonymous	K652K	**C**/T	0.3599	0.3771	108/390/344	126/368/328	0.2863	0.93 (0.81–1.07)
*CDH3*	rs2296408	Synonymous	T271T	**G**/T	0.3698	0.3724	107/394/321	130/388/352	0.8768	1.00 (0.87–1.15)
*CDH3*	rs2296409	Synonymous	T240T	**C**/T	0.3585	0.3643	106/391/344	130/387/371	0.7962	0.98 (0.85–1.13)
*CDH3*	rs8049247	Synonymous	I204I	**A**/C	0.1665	0.1682	21/238/582	22/249/600	0.8683	0.97 (0.81–1.17)
*DACT1*	rs17832998	Missense	A464V	C/**T**	0.3468	0.3448	111/362/369	116/381/392	0.9293	1.01 (0.88–1.17)
*DACT1*	rs863091	Synonymous	V378V	C/**T**	0.2047	0.2033	30/283/525	41/249/524	0.932	1.01 (0.85–1.19)
*HDAC9*	rs1178127	Missense	P621P	A/**G**	0.21	0.2203	37/273/516	41/300/526	0.4737	0.94 (0.80–1.12)
*HDAC9*	rs34096894	Synonymous	L152L	C/**T**	0.01953	0.01351	0/33/812	1/22/865	0.2075	1.33 (0.78–2.27)
*NLK*	rs3182380	Synonymous	I498I	C/**T**	0.05142	0.05535	2/83/761	3/85/734	0.4686	0.92 (0.68–1.24)
*PPARD*	rs2076167	Synonymous	N163N	A/**G**	0.2956	0.294	72/355/417	78/328/417	0.9891	1.00 (0.86–1.16)
*SMURF1*	rs219797	Synonymous	S166S	C/**G**	0.4452	0.4712	160/428/252	210/415/261	0.1591	0.90 (0.78–1.03)
*TCF7*	rs30489	Missense	G256R	C/**T**	0.07683	0.07937	6/118/722	6/128/748	0.7655	0.97 (0.75–1.25)
*TLE1*	rs2228173	Synonymous	E118E	**A**/G	0.1183	0.1172	11/178/656	6/196/685	0.992	1.02 (0.82–1.26)
*WIF1*	rs7301320	Synonymous	A73A	C/**T**	0.2237	0.2219	48/265/494	47/281/517	0.9768	1.00 (0.84–1.18)
*WNT2B*	rs910697	Synonymous	Q390Q	A/**G**	0.4218	0.4301	154/404/286	172/419/296	0.5463	0.95 (0.83–1.09)

Minor allele is depicted in bold.

MAF. Minor Allele Frequency; OR 95% CI. Odds Ratio and 95% Confidence Interval. GT counts. Genotype counts.

*Described OR (95%CI) for rs4444235 and rs9929218 were 1.11 (1.08–1.15) and 0.91 (0.89–0.94), respectively, as taken from Houlston et al.. Nat Genet 2008.

**Table 2:** Association values for stratified analysis in familial and sporadic CRC groups.

		Familial vs control		Sporadic vs control		Familial vs sporadic	
*ADAR*	rs2229857	0.08586	1.28 (0.97–1.68)	0.8662	1.01 (0.87–1.18)	0.1011	1.26 (0.95–1.67)
*APC*	rs2229992	0.6564	1.06 (0.81–1.39)	0.2732	0.92 (0.80–1.07)	0.3214	1.15 (0.87–1.51)
*APC*	rs351771	0.3266	1.15 (0.87–1.50)	0.8956	1.01 (0.87–1.17)	0.3659	1.14 (0.86–1.49)
*APC*	rs41115	0.4254	1.12 (0.85–1.47)	0.9802	1.00 (0.86–1.15)	0.4306	1.12 (0.85–1.47)
*APC*	rs42427	0.3978	1.13 (0.86–1.48)	0.9322	0.99 (0.86–1.15)	0.3825	1.13 (0.86–1.49)
*APC*	rs459552	0.05147	1.35 (1.00–1.83)	0.4821	1.06 (0.90–1.26)	0.1313	1.27 (0.93–1.72)
*APC*	rs465899	0.3161	1.15 (0.88–1.51)	0.8003	1.02 (0.88–1.18)	0.3885	1.13 (0.86–1.49)
*APC*	rs866006	0.3634	1.14 (0.86–1.49)	0.8589	0.99 (0.85–1.14)	0.3256	1.15 (0.87–1.52)
*AXIN1*	rs1805105	0.0674	0.77 (0.58–1.02)	0.5492	0.96 (0.83–1.10)	0.1524	0.81 (0.61–1.08)
*AXIN1*	rs214250	0.5041	1.12 (0.81–1.55)	0.2312	1.11 (0.93–1.32)	0.9975	1.00 (0.72–1.39)
*AXIN1*	rs214252	0.4511	1.13 (0.82–1.57)	0.1736	1.13 (0.95–1.34)	0.9984	1.00 (0.72–1.39)
*AXIN1*	rs400037	0.1971	1.25 (0.89–1.74)	0.6545	0.96 (0.80–1.15)	0.1447	1.29 (0.92–1.81)
*AXIN2*	rs2240308	0.7901	1.04 (0.78–1.36)	0.0733	1.14 (0.99–1.31)	0.5069	0.91 (0.69–1.20)
*BMP4*	rs17563	0.1037	1.25(0.95–1.64)	0.9434	1.01 (0.87–1.16)	0.1119	1.25 (0.95–1.64)
*BMP4*	rs4444235	0.2311	0.85 (0.65–1.11)	0.6689	1.03 (0.90–1.19)	0.1486	0.82 (0.62–1.08)
*BTRC*	rs17767748	0.7285	1.10 (0.63–1.93)	0.813	0.96 (0.71–1.31)	0.6361	1.15 (0.65–2.03)
*BTRC*	rs4151060	0.1176	1.52 (0.90–2.57)	0.4741	0.89 (0.64–1.24)	0.04729	1.72 (1.00–2.96)
*CCND1*	rs603965	0.335	0.87 (0.66–1.15)	0.2045	1.10 (0.95–1.26)	0.1203	0.80 (0.61–1.06)
*CDH1*	rs1801552	0.6563	1.07 (0.80–1.41)	0.08919	0.88 (0.76–1.02)	0.1812	1.21 (0.91–1.61)
*CDH1*	rs9929218	0.8686	0.98 (0.73–1.31)	0.6861	0.97 (0.83–1.13)	0.926	1.01 (0.75–1.37)
*CDH3*	rs1126933	0.1283	1.23 (0.94–1.62)	0.7438	0.98 (0.84–1.13)	0.09059	1.27 (0.96–1.67
*CDH3*	rs17715450	0.2767	0.86 (0.65–1.13)	0.4126	0.94 (0.82–1.09)	0.5064	0.91 (0.68–1.21)
*CDH3*	rs2274239	0.1972	0.83 (0.63–1.10)	0.4589	0.95 (0.82–1.10)	0.3649	0.88 (0.66–1.17)
*CDH3*	rs2296408	0.4447	0.90 (0.68–1.19)	0.9386	1.01 (0.87–1.16)	0.4216	0.89 (0.67–1.18)
*CDH3*	rs2296409	0.1256	0.80 (0.60–1.07)	0.9158	1.01 (0.87–1.17)	0.1138	0.79 (0.59–1.06)
*CDH3*	rs8049247	0.9636	1.01 (0.71–1.44)	0.867	0.98 (0.82–1.19)	0.9021	1.02 (0.71–1.47)
*DACT1*	rs17832998	0.9185	0.99 (0.74–1.31)	0.8619	1.01 (0.88–1.17)	0.8392	0.97 (0.73–1.29)
*DACT1*	rs863091	0.5683	0.90 (0.64–1.28)	0.7737	1.03 (0.86–1.22)	0.4595	0.88 (0.62–1.24)
Gene	SNP ID	p-value	OR (CI 95%)	p-value	OR (CI 95%)	p-value	OR (CI 95%)
*HDAC9*	rs1178127	0.8693	1.03 (0.74–1.42)	0.3847	0.93 (0.78–1.10)	0.5511	1.11 (0.80–1.54)
*HDAC9*	rs34096894	0.8555	0.89 (0.27–2.99)	0.1093	1.55 (0.90–2.67)	0.3638	0.58 (0.18–1.91)
*NLK*	rs3182380	0.4747	0.79 (0.42–1.50)	0.7387	0.95 (0.69–1.30)	0.5917	0.84 (0.44–1.60
*PPARD*	rs2076167	0.1051	0.77 (0.57–1.06)	0.5291	1.05 (0.90–1.23)	0.06342	0.74 (0.55–1.02)
*SMURF1*	rs219797	0.9123	0.99 (0.75–1.29)	0.09224	0.89 (0.77–1.02)	0.4764	1.10 (0.84–1.45)
*TCF7*	rs30489	0.1722	1.36 (0.87–2.11)	0.4351	0.90 (0.69–1.17)	0.07095	1.51 (0.96–2.38)
*TLE*	rs2228173	0.4715	1.16 (0.78–1.71)	0.8995	0.99 (0.79–1.23)	0.4626	1.16 (0.78–1.73)
*WIF1*	rs7301320	0.2681	1.20 (0.87–1.64)	0.8226	0.98 (0.83–1.16)	0.2418	1.21 (0.88–1.67)
*WNT2B*	rs910697	0.4228	0.90 (0.68–1.17)	0.7713	0.98 (0.85–1.13)	0.5418	0.92 (0.70–1.21)

MAF. Minor Allele Frequency; OR 95% CI. Odds Ratio and 95% Confidence Interval.

Thus, our strategy has not managed to detect any new susceptibility loci for CRC risk.

Pathway-based expectations have proved to be quite discouraging in the literature as well, for strong candidate pathways, such as DNA-repair ones, surprisingly failed too in identifying any new risk variants [7], [23]–[24]. In addition to this, most of the genetic variants that have been found to be associated with disease are located in intergenic regions, with potential functions that are yet unknown.

Still, in light of the recent discoveries that followed up the analysis of genome-wide data, both Wnt and BMP have earned a renewed fame. The susceptibility locus found on 8q24 (rs6983267) has been linked to an enhanced Wnt signaling through its interaction with TCF4 [25], [26], and a meta-analysis conducted on a series of GWAS data succeeded in associating two variants in the *BMP4* and *CDH1* gene regions with the disease (rs4444235 and rs9929218, respectively)[8].

Even though this is actually the first association study that considers the pathways as a whole for gene selection, some of the genes included in our analysis (i.e *APC, CCND1*, *CDH1* and *TCF7*) had already been screened for risk alleles [6], [27]–[30]. It is quite remarkable that there has been a growing debate over some of these loci, specially the p.V1822D variant in *APC* (rs459552). This missense change is widely documented in the literature, with some studies defending it as neutral (this study and others)[31], and some conferring its minor allele a protective effect [6], [28]. Lack of appropriate study power, resultant from insufficient number of samples has been a major problem in many of these studies and thus most of them have not provided very convincing results [32].

Although our study had over 80% power to detect OR as low as 1.21 with minor allele frequencies of 0.30 (57% of our SNPs), and 1.24 for MAFs down to 0.2 (78% of the SNPs), assuming a log-additive model and α = 0.05, we were unable to detect any positive associations suggesting the presence of any new CRC susceptibility variants. Nevertheless, it is quite remarkable that, albeit our failure to replicate the associations for the *BMP4* and *CDH1* SNPs, this is the first study that investigates any of the so-called 10 new GWAS-discovered susceptibility loci in a Southern-European population.

Despite our negative results, we must consider that we did not whatsoever comprehensively cover all possible low-penetrance variants within the selected genes. This is mainly due to the fact that our strategy was purely functional, selecting the variants that were *a priori* good candidates to be directly associated with the disease. This indeed may constitute a limitation in the study, for most of the genetic variation within the loci was not investigated. Thus, we believe further efforts should be made to screen a wider variety of loci within these pathways, specially considering the previous positive associations described so far for both Wnt and BMP-related genes.

Pondering the potential odds ratios of the variants described so far (1.11, CI 1.08–1.15 and 0.91, CI 0.89–0.94 for rs4444235 and rs9929218, respectively), we assume larger cohorts may be required to detect such subtle effects. On the other hand, when considering candidate-gene approaches, it would also be useful to meta-analyse previous studies and pull the information across of them altogether in the search of evidences of potential new pathways linked to the pathogenesis of the disease.

## Materials and Methods

### Study populations

Subjects were 933 CRC patients and 969 controls that belonged to the EPICOLON project, a prospective, multicentre, population-based epidemiology survey studying the incidence and features of familial and sporadic CRC in the Spanish population [33]. Cases were selected across 11 hospitals in Spain as all patients with a *de-novo* histologically confirmed diagnosis of colorectal adenocarcinoma and who attended 11 community hospitals across Spain between November 2006 and December 2007. Patients in whom CRC developed in the context of familial adenomatous polyposis or inflammatory bowel disease, and cases where patients or family refused to participate in the study were excluded. Demographic, clinical and tumour-related characteristics of probands, as well as a detailed family history were obtained using a pre-established questionnaire, and registered in a single database. Of these, 592 (63%) were male and 341 (37%) female. Median age for cases was 73 (range 26–95), whereas mean was 71(SD±10.7). Hospital-based controls were recruited together with cases and were confirmed to have no cancer or prior history of neoplasm, and no family history of CRC. All controls were randomly selected and matched with cases for sex and age (±5 years) in a 1∶1 ratio. Both cases and controls were of European ancestry and from Spain.

### Ethics statement

The study was approved by the “Comité Ético de Investigación Clínica de Galicia”, and each of the institutional review boards of the hospitals where samples were collected (“Ethics Committee of the Hospital Clínic-Barcelona”, “Ethics Committee of the Hospital del Mar-Barcelona”, “Ethics Committee of the Hospital German Trias i Pujol-Barcelona”, “Ethics Committee of the Hospital Sant Pau-Barcelona,” “Ethics Committee of the Hospital Universitari Arnau de Vilanova-Lleida”, “Ethics Committee of the Hospital General-Alicante”, “Ethics Committee of the Hospital de Donosti”, “Ethics Committee of the Hospital General de Asturias-Oviedo”, “Ethics Committee of the Hospital Clinico-Zaragoza”, “Ethics Committee of the Hospital de Calahorra-La Rioja”, “Ethics Committee of the Hospital Meixoeiro-Vigo”). All samples were obtained with written informed consent reviewed by the ethical board of the corresponding hospital.

### DNA extraction

DNA was obtained from frozen peripheral blood; extraction was performed in a CHEMAGEN robot (Chemagen Biopolymer-Technologie AG, Baesweiler, Germany) in accordance with the manufacturer's instructions, at the Galician Public Fundation of Genomic Medicine in Santiago de Compostela. Cases and controls were extracted in mixed batches to avoid any kind of bias.

### Candidate-gene selection

Both Wnt and BMP pathways were initially selected after the findings of Nishanian et al. [34], who demonstrated the interaction between these two pathways. Both pathways were thoroughly investigated through the Cancer Genome Anatomy Project site [35], but we failed to find any information regarding the BMP pathway in either this or other web browsers. For that reason, Wnt genes were selected by browsing the pathway through Biocarta [36], whereas BMP genes had to be strictly selected from previous literature [17], [34]. Forty-one genes were finally selected to be included in the analysis.

### SNP selection and genotyping

SNP selection criteria only considered functional markers with minor allele frequencies above 0.05 and at least two independent validation criteria as established in dbSNP [37]. This included all exonic variants selected with Pupasuite [38] and gene-regulatory regions in *cis* (5′or 3′ UTR ends), as defined by the FESD web browser [39]. 5′UTR variants were only included when they complied to the abovementioned criteria and were presumed to be in the potential binding site of a known transctiptional binding factor. 3′ UTR variants were included because of their potential relationship with miRNA binding regions [40]. Because some of the selected genes had no SNPs of such these kinds in any of the three browsers at the time of SNP selection, they ultimately had to be dropped out of the study. Finally, 43 SNPs were chosen within 21 genes to be screened as potential direct modifiers of CRC susceptibility (Table 3).

**Table 3:** Description of all genes selected from both pathways and SNPs screened within each of them.

Gene Name	Function	pathway/genes modulated by BMP signalling	SNPs selected
***ADAR,*** ** Adenosine deaminase, RNA- specific**	Converts multiple adenosines to inosines and creates I/U mismatched base pairs in double-helical RNA	Wnt signalling^36^	rs2229857
***APC,*** ** Adenomatous Polyposis Coli**	B-catenin degradation	Wnt signalling^36^	rs2229992,rs351771,rs4115,rs42427rs459552,rs465899,rs86006
***AXIN1*** **, Axin 1**	B-catenin regulation	Wnt signalling^36^	rs1048786,rs1805105,rs214250,rs214252,rs400037,rs419949
***BTRC*** **, Beta-transducin repeat containing**	B-catenin ubiquitination	Wnt signalling^36^	rs17767748,rs415060
***CCND1*** **, Cyclin D1**	Cell cycle control	Wnt signalling^36^	rs603965
*CSNK1A1*, Casein kinase 1, alpha 1	B-catenin fosforilation	Wnt signalling^36^	NA
*CSNK2A1*, Casein kinase 2, alpha 1	B-catenin fosforilation	Wnt signalling^36^	NA
*CTBP1*, C-terminal binding protein 1	Transcriptional repressor in cellular proliferation	Wnt signalling^36^	NA
*CTNNB1*, Catenin (cadherin-associated protein), beta 1	Cell adhesion and signal transduction	Wnt signalling^36^	NA
*EIF4E*, Eukaryotic translation initiation factor 4E	Translation initiation factor	Wnt signalling^36^	NA
*ELAC1*, ElaC homolog 1 (E. coli)	Zinc phosphodiesterase	Wnt signalling^36^	NA
*FRAT1*, Frequently rearranged in advanced T-cell lymphomas	B-catenin stabilization	Wnt signalling^36^	NA
*FZD1*, Frizzled homolog 1 (Drosophila)	Receptor for Wnt proteins	Wnt signalling^36^	NA
***GSK3B*** **, Glycogen synthase kinase 3 beta**	B-catenin fosforilation	Wnt signalling^36^	rs34002644
***HDAC9*** **, Histone deacetylase 9**	Transcriptional regulation, cell cycle	Wnt signalling^36^	rs1178127,rs34096894
***HNF4A*** **, Hepatocyte nuclear factor 4, alpha**	Transcriptionally controlled transcription factor	Wnt signalling^36^	rs35078168
*MAP3K7*, Mitogen-activated protein kinase kinase kinase 7	Signaling transduction induced by BMP	Wnt signalling^36^	NA
*MYC*, v-myc myelocytomatosis viral oncogene homolog (avian)	Regulation of gene transcription	Wnt signalling^36^	NA
***NLK*** **, Nemo-like kinase**	Negatively regulation wnt pathway	Wnt signalling^36^	rs3182380
***PPARD*** **, Peroxisome proliferator-activated receptor delta**	Ligand-activated transcription factor.	Wnt signalling^36^	rs2076167
*PPP2R4,* Protein phosphatase 2A activator, regulatory subunit 4	Folding of proteins	Wnt signalling^36^	NA
***TLE1*** **, Transducin-like enhancer of split 1 (E(sp1) homolog, Drosophila)**	Transcriptional corepressor	Wnt signalling^36^	rs2228173,rs8782
***WIF1*** **, Wnt inhibitory factor 1**	Inhibition of the WNT activities	Wnt signalling^36^	rs1026024,rs7301320
*WNT1*, Wingless-type MMTV integration site family, member 1	Ligand for members of the frizzled family	Wnt signalling^36^	NA
***BMP4*** **, Bone morphogenetic protein 4**	Induces cartilage and bone formation.	BMP signalling^17^	rs17563
*BMPR1B*, Bone morphogenetic protein receptor, type IB	Transmembrane serine/threonine	BMP signalling^17^	NA
*SMAD1*, SMAD family member 1	Signal transduction	BMP signalling^17^	NA
***SMAD4*** **, SMAD family member 4**	Signal transduction	BMP signalling^17^	rs75667697
*SMAD5*, SMAD family member 5	Signal transduction	BMP signalling^17^	NA
***SMURF1*** **, SMAD specific E3 ubiquitin protein ligase 1**	Ubiquitination and degradation of SMAD proteins	BMP signalling^17^	rs219797
***AXIN2*** **, Axin 2**	B-catenin regulation	Wnt signalling, BMP induced genes^34^	rs2240308
***CDH1*** **, Cadherin 1, type 1, E-cadherin**	B-catenin regulation	Wnt signalling, BMP induced genes^34^	rs1801552
***CDH3*** **, Cadherin 3, type 1, P-cadherin (placental)**	B-catenin regulation	Wnt signalling, BMP induced genes^34^	rs1126933,rs17715450,rs2274239,rs2296408,rs2296409,rs8049247
*DAB2*, Disabled homolog 2, mitogen-responsive phosphoprotein	B-catenin regulation	Wnt signalling, BMP induced genes^34^	NA
***DACT1*** **, Dapper antagonist of beta-catenin, homolog 1 (Xenopus laevis)**	Disheveled inhibitor	Wnt signalling, BMP induced genes^34^	rs17832998,rs698025,rs863091
*KIFAP3*, Kinesin-associated protein 3	Interacts with apc	Wnt signalling, BMP induced genes^34^	NA
*LEF1*, Lymphoid enhancer-binding factor 1	Transcriptional activator of Wnt signaling	Wnt signalling, BMP induced genes^34^	NA
***TCF7*** **, Transcription factor 7 (T-cell specific, HMG-box)**	transcriptional repressor of CTNNB1	Wnt signalling, BMP induced genes^34^	rs30489
***WNT2B*** **, Wingless-type MMTV integration site family, member 2B**	Wnt ligand	Wnt signalling, BMP induced genes^34^	rs910697
*WNT5A*, Wingless-type MMTV integration site family, member 5A	Wnt ligand	Wnt signalling, BMP induced genes^34^	NA
*WNT5B*, Wingless-type MMTV integration site family, member 5B	Wnt ligand	Wnt signalling, BMP induced genes^34^	NA

Genes finally screened are depicted in bold.

NA denotes not available SNPs for a given gene considering our selection criteria. rs4444235 and rs9929218 are not shown, for they were included because of their previous associations and not because they fulfilled our functional criteria.

**Figure 1:**
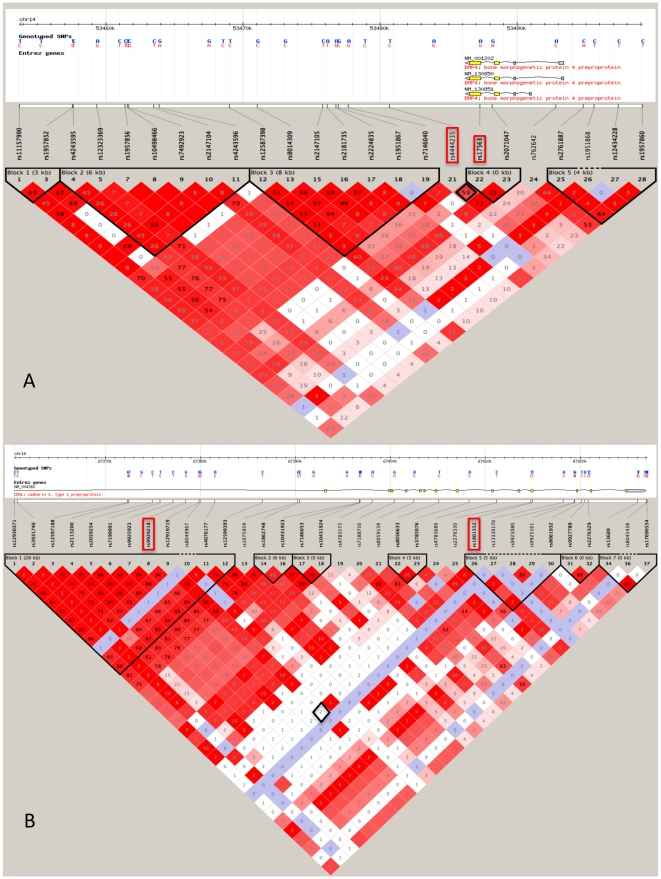
Linkage disequilibrium blocks for the *BMP4* and *CDH1* genes. R-squared relationships between SNP pairs: A. rs4444235-rs17563 in *BMP4* and B. rs9929218-rs1801552 in *CDH1*.

rs4444235 and rs9929218 are two variants lying in the near-by and intronic regions of *BMP4* and *CDH1*, respectively, that have been recently reported to be associated with the disease [8]. Considering that the SNPs that we had chosen within these two genes were not good taggers for these two variants (r-squared values were 0.6 for the SNPs in *BMP4*, and 0.02 for those in *CHD1*) (Figure 1), we decided to include them in our study as well, although they did not fulfill our selection criteria, making the total number of interrogated SNPs rise to 45.

Genotyping was performed with the MassARRAY (Sequenom Inc., San Diego, USA) technology at the Santiago de Compostela node of the Spanish Genotyping Center. Calling of genotypes was done with Sequenom Typer v4.0 software using all the data from the study simultaneously.

### Statistical analyses

Quality control was performed, first by excluding both SNPs and samples with genotype success rates below 95%, with the help of the Genotyping Data Filter (GDF) [41]. Genotypic distributions for all SNPs in controls were consistent with Hardy-Weinberg equilibrium as assessed using a X^2^ test (1^df^). All p-values obtained were ≥0.05, thereby excluding the possibility of genotyping artifacts (data not shown). Population stratification was assessed with Structure v2.2 [42]. Briefly, the posibility of different scenarios was tested assuming a different number of underlying populations (k ranging from 1 to 4), allowing for a large number of iterations (25 K in the burn-in period followed by 500 K repetitions). The mean log likelihood was estimated for the data for a given k (referred to as L(K)) in each run. We as well performed multiple runs for each value of k computing the overall mean L(K) and its standard deviation. All results seemed to be concordant with the original assumption of a single existing population. Moreover, additional procedures for better confounding variable visualization were undertaken by means of a Principal Component Analysis (PCA) using the EIGENSOFT tool *smartpca* [43], although number of markers was very low. No differences were found of population stratification between cases and controls for either STRUCTURE or the first 10 components of the PCA analysis (Figure S2). After quality control 1746 samples (854 cases and 892 controls) and 37 SNPs remained for further analyses.

Association tests were performed by chi-squared tests for every single SNP and haplotypes where possible with both Haploview v4.0 [44] and Unphased [45]. In short, LD patterns across genes for which more than one SNP was genotyped were checked in Haploview and tested for association using Unphased (to check in any of the haplotypes was associated) and Haploview (to see which of the haplotypes was associated). Genotypic association tests, logistic regression analysis for sex and age adjustment, and stratified analysis between sporadic and familial groups were estimated with PLINK v1.03 [46]. OR and 95% confidence intervals were calculated for each statistic, and to address the issue of multiple-testing, permutation tests and the Bonferroni correction were used. Study power was estimated with CATS software [47].

## Supporting Information

Figure S1 Haplotype structure and analysis for the 8 genes for which more than one SNP was genotyped. The table shows association values for each SNP generated by Haploview.
(3.40 MB TIF)

Figure S2 Principal component analysis plot for the first vs. second component, comparing our case and control populations.
(0.96 MB TIF)

Note S1 (0.03 MB DOC)
