# Supplementary figures and images for: Single Nucleotide Polymorphisms in the Wnt and BMP Pathways and Colorectal Cancer Risk in a Spanish Cohort
Source: PLoS One. 2010 Sep 9;5(9):e12673. doi: 10.1371/journal.pone.0012673 (PMC2936577; doi:10.1371/journal.pone.0012673)

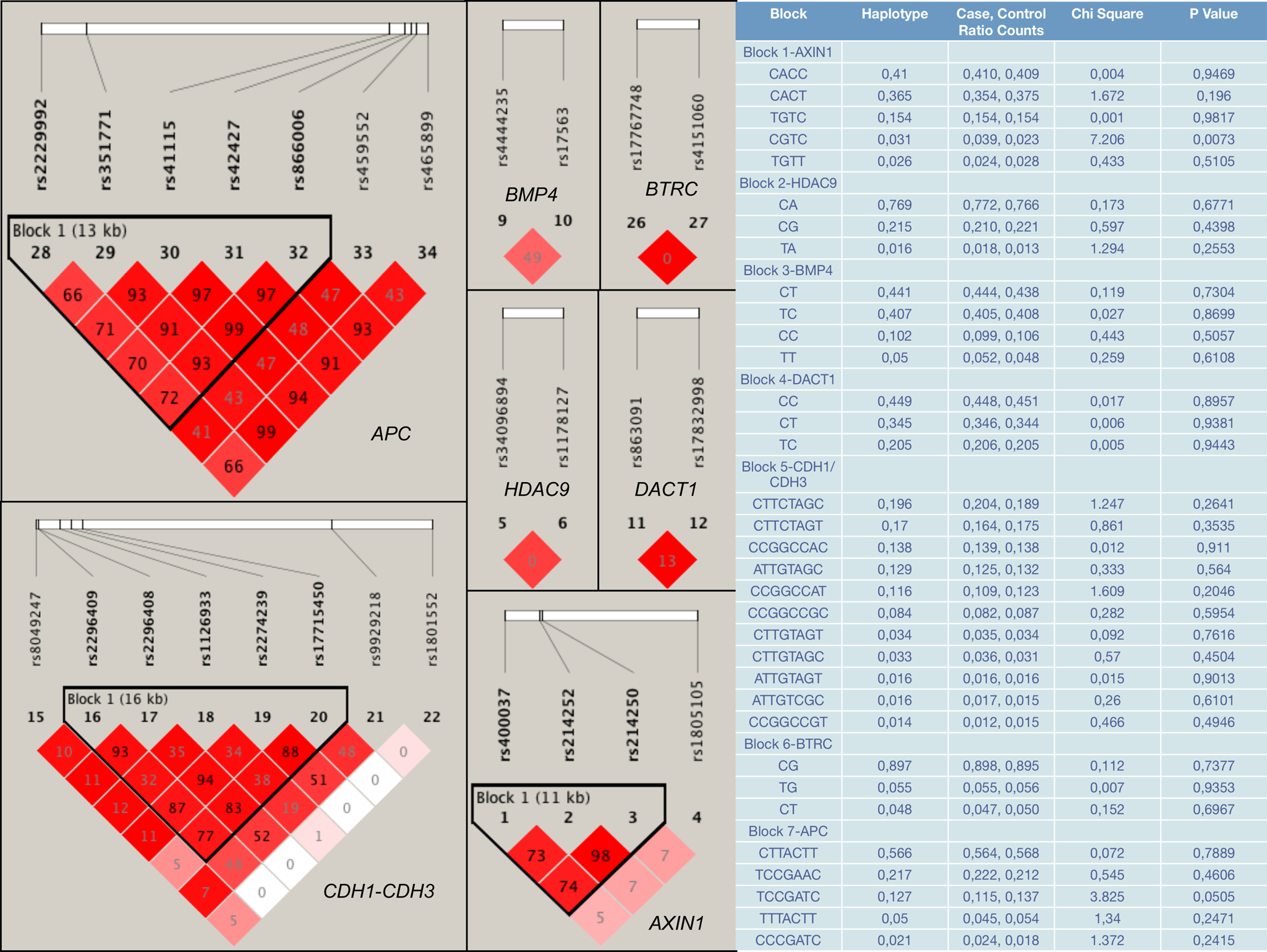

Supplement: Figure S1 — Haplotype structure and analysis for the 8 genes for which more than one SNP was genotyped. The table shows association values for each SNP generated by Haploview. (3.40 MB TIF) [file pone.0012673.s001.tif]

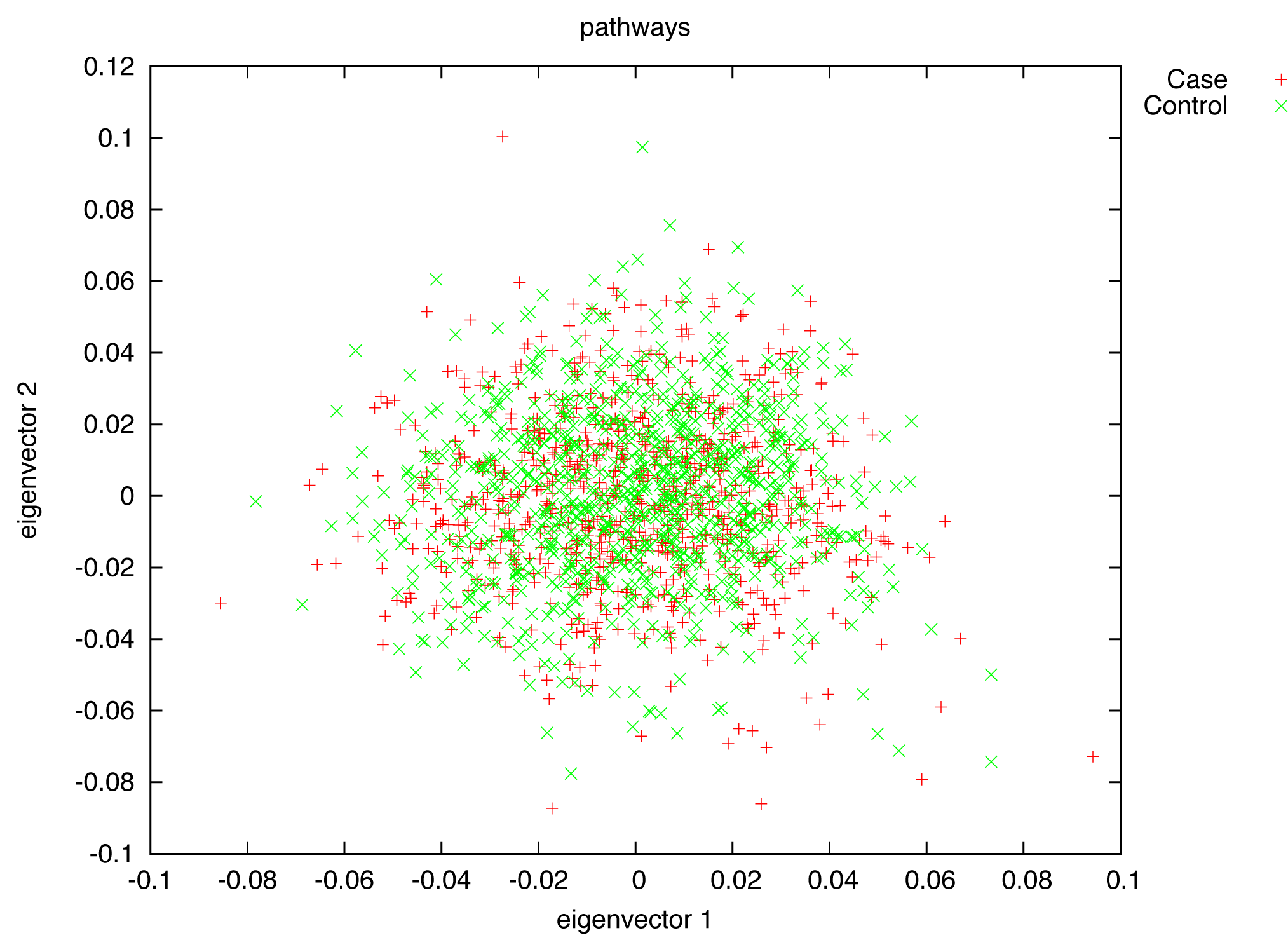

Supplement: Figure S2 — Principal component analysis plot for the first vs. second component, comparing our case and control populations. (0.96 MB TIF) [file pone.0012673.s002.tif]
