# Supplementary material for: Single Nucleotide Polymorphisms in the Wnt and BMP Pathways and Colorectal Cancer Risk in a Spanish Cohort
Source: PLoS One. 2010 Sep 9;5(9):e12673. doi: 10.1371/journal.pone.0012673 (PMC2936577; doi:10.1371/journal.pone.0012673)
Supplement: Note S1 — (0.03 MB DOC) [file pone.0012673.s003.doc]

**Supplementary note. Members of the EPICOLON Consortium (Gastrointestinal Oncology Group of the Spanish Gastroenterological Association)**

Hospital 12 de Octubre, Madrid: Juan Diego Morillas (local coordinator), Raquel Muñoz, Marisa Manzano, Francisco Colina, Jose Díaz, Carolina Ibarrola, Guadalupe López, Alberto Ibáñez; Hospital Clínic, Barcelona: Antoni Castells (local coordinator), Virgínia Piñol, Sergi Castellví-Bel, Francesc Balaguer, Victoria Gonzalo, Teresa Ocaña, María Dolores Giráldez, Maria Pellisé, Anna Serradesanferm, Leticia Moreira, Miriam Cuatrecasas, Josep M. Piqué; Hospital Clínico Universitario, Zaragoza: Ángel Lanas (local coordinator), Javier Alcedo, Javier Ortego; Hospital Cristal-Piñor, Complexo Hospitalario de Ourense: Joaquin Cubiella (local coordinator), Mª Soledad Díez, Mercedes Salgado, Eloy Sánchez, Mariano Vega; Hospital del Mar, Barcelona: Montserrat Andreu (local coordinator), Anna Abuli, Xavier Bessa, Mar Iglesias, Agustín Seoane, Felipe Bory, Gemma Navarro, Beatriz Bellosillo; Josep Mª Dedeu, Cristina Álvarez, Begoña Gonzalez; Hospital San Eloy, Baracaldo and Hospital Donostia, San Sebastián: Luis Bujanda (local coordinator) Ángel Cosme, Inés Gil, Mikel Larzabal, Carlos Placer, María del Mar Ramírez, Elisabeth Hijona, Jose M. Enríquez-Navascués y Jose L. Elosegui;  Hospital General Universitario de Alicante: Rodrigo jover (local coordinator): Artemio payá, Cristina alenda,  Laura Sempere,  Nuria Acame, Estefanía Rojas, Lucía Pérez-Carbonell; Hospital General de Granollers: Joaquim Rigau (local coordinator), Ángel Serrano, Anna Giménez; Hospital General de Vic: Joan Saló (local coordinator), Eduard Batiste-Alentorn, Josefina Autonell, Ramon Barniol; Hospital General Universitario de Guadalajara and Fundación para la Formación e Investigación Sanitarias Murcia: Ana María García (local coordinator), Fernando Carballo, Antonio Bienvenido, Eduardo Sanz, Fernando González, Jaime Sánchez, Akiko Ono; Hospital General Universitario de Valencia: Enrique Medina (local coordinator), Mercedes Latorre, Jaime Cuquerella, Pilar Canelles, Miguel Martorell, José Ángel García, Francisco Quiles, Elisa Orti; CHUVI-Hospital Meixoeiro, Vigo: EPICOLON I: Juan Clofent (local coordinator), Jaime Seoane, Antoni Tardío, Eugenia Sanchez. EPICOLON II Mª Luisa de Castro (local coordinator), Antoni Tardío, Juan Clofent , Vicent Hernández; Hospital Universitari Germans Trias i Pujol, Badalona and Section of Digestive Diseases and Nutrition, University of Illinois at Chicago, IL, USA. : Xavier Llor (local coordinator), Rosa M. Xicola, Marta Piñol, Mercè Rosinach, Anna Roca, Elisenda Pons, José M. Hernández, Miquel A. Gassull; Hospital Universitari Mútua de Terrassa: Fernando Fernández-Bañares (local coordinator), Josep M. Viver, Antonio Salas, Jorge Espinós, Montserrat Forné, Maria Esteve; Hospital Universitari Arnau de Vilanova, Lleida: Josep M. Reñé (local coordinator), Carmen Piñol, Juan Buenestado, Joan Viñas; Hospital Universitario de Canarias: Enrique Quintero (local coordinator), David Nicolás, Adolfo Parra, Antonio Martín; Hospital Universitario La Fe, Valencia: Lidia Argüello (local coordinator), Vicente Pons, Virginia Pertejo, Teresa Sala; Hospital Sant Pau, Barcelona: Dolors Gonzalez (local coordinator) Eva Roman, Teresa Ramon, Maria Poca, Mª Mar Concepción, Marta Martin, Lourdes Pétriz; Hospital Xeral Cies, Vigo: Daniel Martinez (local coordinator); Fundacion Publica Galega de Medicina Xenomica (FPGMX), CIBERER, Genomic Medicine Group-University of Santiago de Compostela, Santiago de Compostela, Galicia, Spain: Angel Carracedo (local coordinator), Clara Ruiz-Ponte, Ceres Fernández-Rozadilla, María J Magdalena-Castro; Hospital Universitario Central de Asturias: Sabino Riestra (local coordinator), Luis Rodrigo; Hospital de Galdácano, Vizcaya: Javier Fernández (local coordinator), Jose Luis Cabriada; Fundación Hospital de Calahorra, La Rioja: Luis Carreño (local coordinator).
